# Control theory for scanning probe microscopy revisited

**DOI:** 10.3762/bjnano.5.38

**Published:** 2014-03-21

**Authors:** Julian Stirling

**Affiliations:** 1 School of Physics and Astronomy, The University of Nottingham, University Park, Nottingham, NG7 2RD, United Kingdom

**Keywords:** AFM, control theory, feedback, scanning probe microscopy

## Abstract

We derive a theoretical model for studying SPM feedback in the context of control theory. Previous models presented in the literature that apply standard models for proportional-integral-derivative controllers predict a highly unstable feedback environment. This model uses features specific to the SPM implementation of the proportional-integral controller to give realistic feedback behaviour. As such the stability of SPM feedback for a wide range of feedback gains can be understood. Further consideration of mechanical responses of the SPM system gives insight into the causes of exciting mechanical resonances of the scanner during feedback operation.

## Introduction

Scanning probe microscopy (SPM) imaging relies on feedback loops to maintain a constant interaction between the tip and the sample [1–2]. Many well known artefacts can arise from improper feedback settings [3–5]. Thus, for reliable SPM operation and analysis the characteristics and behaviour of such feedback loops must be considered [6–7]. SPM feedback loops usually employ a proportional-integral (PI) controller, equivalent to the common proportional-integral-differential (PID) controller with the differential gain set to zero to avoid amplification of noise. Other groups have successfully modelled and implemented proportional-differential controllers [8], but these are not commonly used. Previous work has used control theory to analyse the behaviour of PI and PID feedback loops in the context of SPM [9–12], and these models are still being applied in the current literature [13]. However, the details of the operation of the feedback loop have been incorrectly modelled, which results in a decreased stability and an exaggerated ringing at the resonant frequency of the piezoelectric actuator (*z*-piezo). Due to these errors, the feedback controller often cannot maintain tracking without a high derivative component [13], which is entirely at odds with experimental observations. This paper employs analysis of specific SPM PI controllers to provide a more appropriate method for modelling such systems.

## Results and Discussion

**Figure 1:**
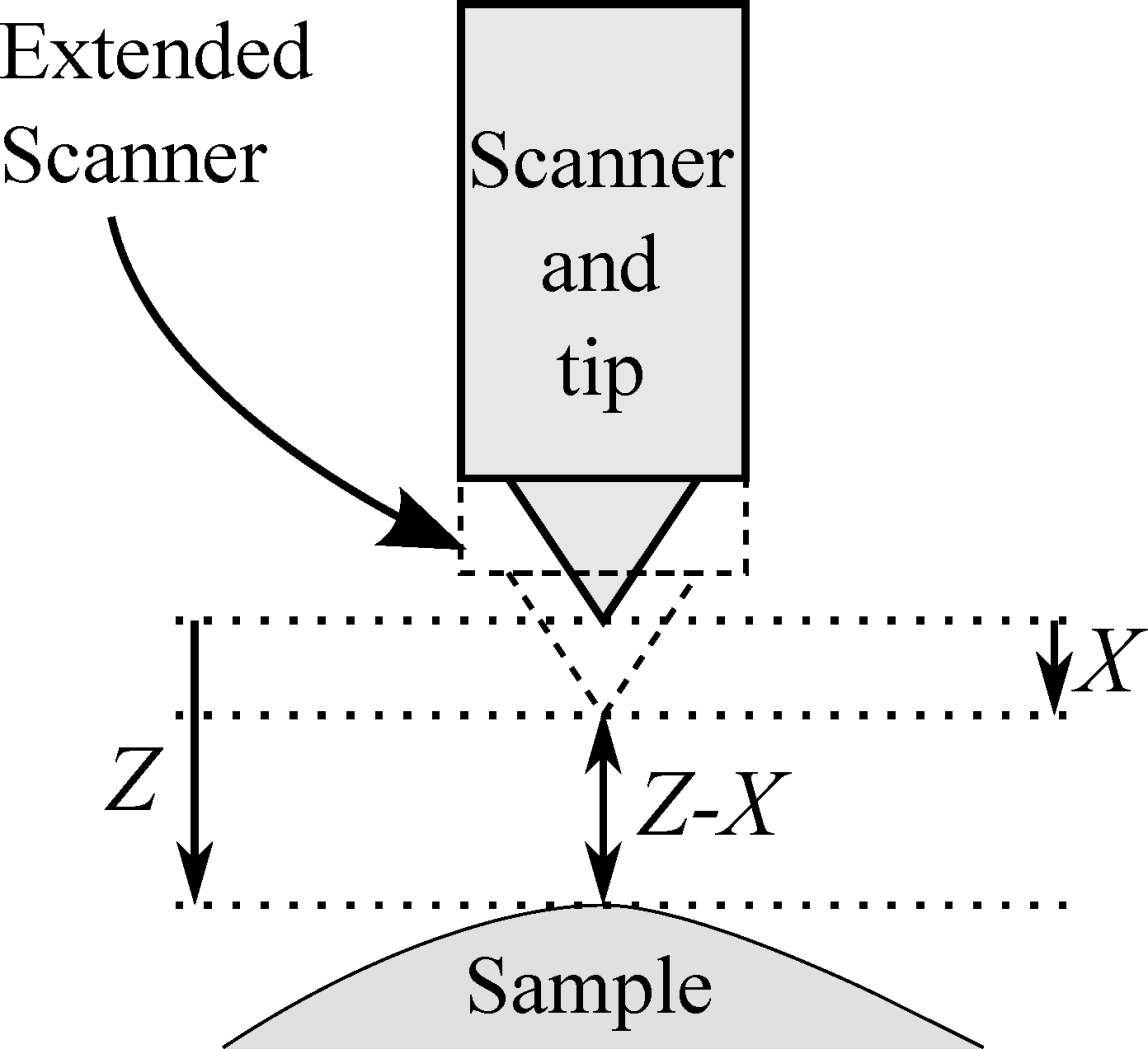
Schematic showing coordinates for sample position, *Z*, and scanner extension, *X*. The tip–sample distance can then be calculated as *Z* – *X*.

When modelling an SPM feedback loop we must first consider the workings of the PI controller under perfect conditions. First, assume that the tip is stationary above a sample at a position *Z*, and that the *z*-piezoelectric actuator for tip positioning is extended by *X* (Figure 1). For this perfect model *X* is considered to be directly the output of the PI controller; consideration of amplifier bandwidths and mechanical resonances is added later. For our original simplified model we will consider a generic SPM which tracks to a set-point tip–sample distance (Note that the exact mechanism to detect this distance is not relevant). Referring to the set-point distance as *P*, and the tip–sample distance as *Z* – *X*, then the error signal input to the PI controller, *E*, is equal to

[1]
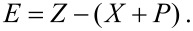


After a time *t* in feedback the output of a standard PI controller would be

[2]
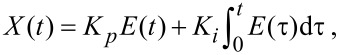


where *K_p_* and *K_i_* are the proportional and integral gains of the PI controller respectively and τ is a dummy integration variable. For this standard PI controller the output of the first term is proportional to the instantaneous error, and the output of the second term is proportional to the error that was integrated since the start of the experiment. It is clear that such a system is intrinsically unstable, by considering the case that *E*(*t*_0_) = 0. As the tip–sample distance is equal to the set-point distance there should be no movement. However, evaluating Equation 2 the output to the piezo *X*(*t*_0_ + d*t*) will be zero (where the d*t* is used to clarify that the system was not initiated at 0 but the first output after initiation will be zero.). Thus, the tip will return to the zero piezo extension position, rather than stay static (because the error signal is zero). At the next time step, there will be a large error signal and the tip will move back towards its correct position. This rudimentary problem has apparently gone unnoticed to date because it has been ‘disguised’ by the more complicated modelling of the response of the various other electrical and electromechanical components of the SPM (amplifiers, piezoelectric actuators).

It is helpful to draw an analogy with the most commonly considered control system, namely a temperature controller. A conventional PI controller in essence calculates the heat to be added to the system under control. If the set-point matches the measured temperature an output of zero is required. However, an SPM directly controls the extension of the piezoelectric actuator, which is analogous to directly controlling the temperature. To correct for this one must consider that the output of a PI controller in an SPM is the *change* in the extension. Thus, for the final output of the feedback controller to be the extension we must integrate the PI controller output since the start of the experiment (with *X*(0) = 0):

[3]
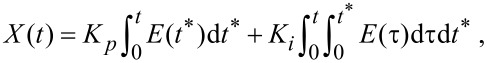


where t^*^ is another dummy integration variable. This integration effectively stores all previous feedback response. Comparing to Equation 2 we see that if initiated under the same conditions, where *X*(0) = 0, the integral term does store previous response as a proportional controller (i.e., the second term of Equation 2 is equivalent to the first term of Equation 3). Thus, the controller implemented by Equation 2 would perform as a proportional-differential controller.

**Figure 2:**
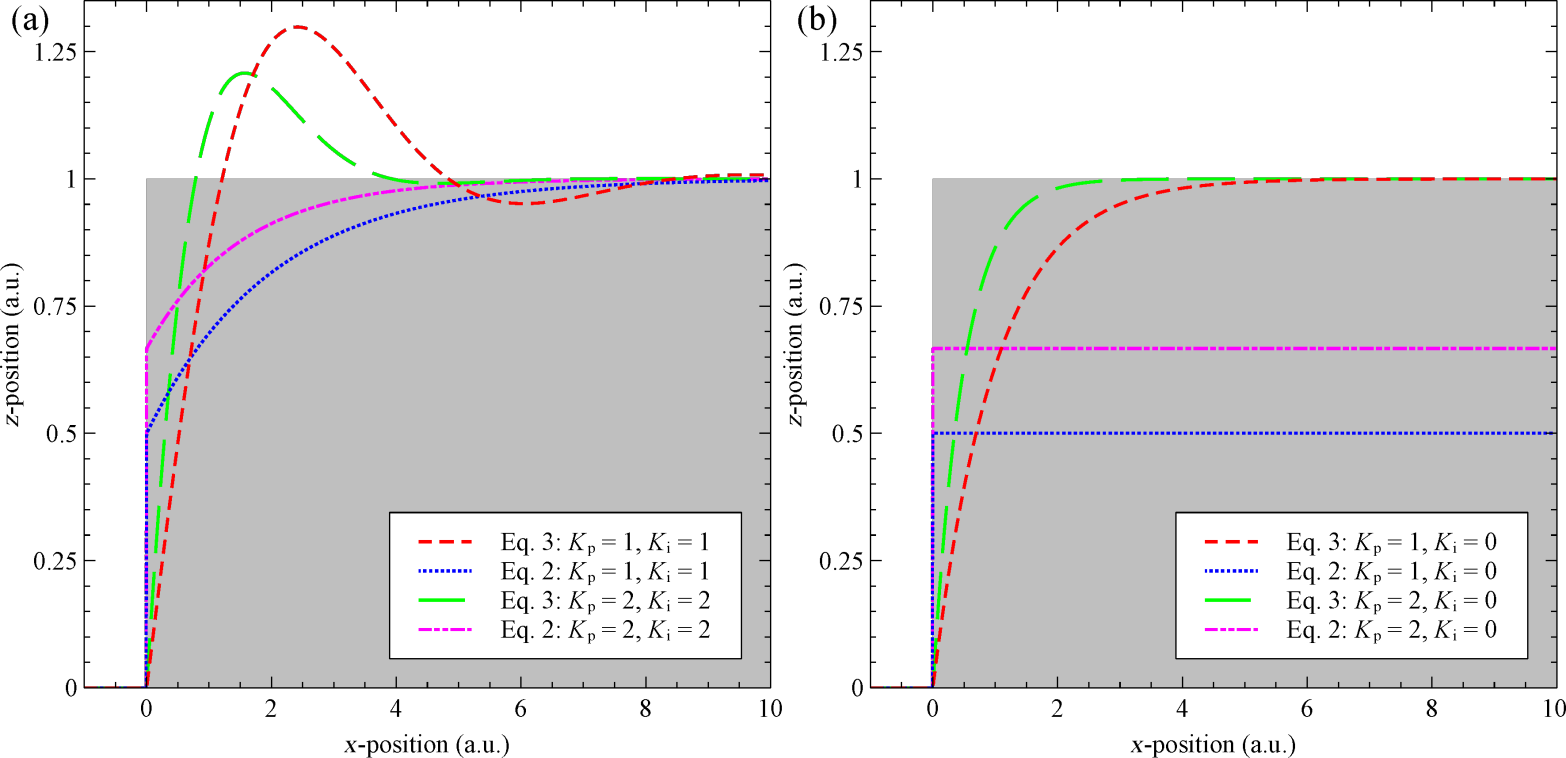
Direct comparison of our model (Equation 3, red and green lines) with the model from the current literature (Equation 2, blue and pink lines), without modelling of electrical or mechanical components. The comparison is performed for a full PI controller (a) and a simple proportional controller (b), where the grey area represents the surface being tracked with a set-point of 0. Equation 2 shows unexpected discontinuities and does not tract the set-point for a proportional controller. Instead it only reacts to the initial impulse. Equation 3 produces the expected results from elementary control theory. All gain units are arbitrary.

Figure 2 directly compares the response of Equation 2 and Equation 3 to a unit step, analytically solved by using a Laplace transform with a set-point of zero. For a PI controller, Figure 2a, modelled by using Equation 2 there is a discontinuity in the extension at the time of the step, this results from the incorrectly modelled proportional controller that acts as a derivative controller. This discontinuity can go unnoticed if the equations are solved numerically, if a frequency cut-off is modelled [9], or if the mechanical response of the *z*-piezo is modelled. Additionally, the controller modelled by using Equation 2 does not experience the expected overshoot of the set-point for a PI controller, this can also go unnoticed when mechanical response of the *z*-piezo is modelled as its resonance can be mistaken for feedback ringing [9]. By further examining Equation 2 for a proportional controller (*K_i_* = 0), we see (Figure 2b) that in addition to the discontinuity the controller settles to a value that is a 1/(*K_p_* + 1) of the required extension. This has previously been mistaken as a steady-state error common to proportional controllers [9]. However, when plotted without any modelling of other components it becomes clear that it results from the controller that only acts to the initial impulse.

From Figure 2b it becomes apparent that there will be no steady-state offset when evaluating the response of Equation 3 to a static surface (*Z*(*t*) = *E* + *X* + *P* = constant), for a simple proportional controller (*K_i_* = 0). This initially appears at odds with both experiments and elementary control theory. However, this is due to the simplicity of the system we are modelling. Again considering our analogous temperature controller it is well known that the cause of the steady-state error is the fact that the heat input into the system is equal to the heat lost to (or gained from) outside the system. Now we see that steady-state errors in SPM feedback result from a sample drift in the *z*-direction or from scanning a sample with a tilt. Thus, any system that does not model *z*-drift or sample tilt should not expect a steady-state error.

### Complete model of SPM feedback

**Figure 3:**
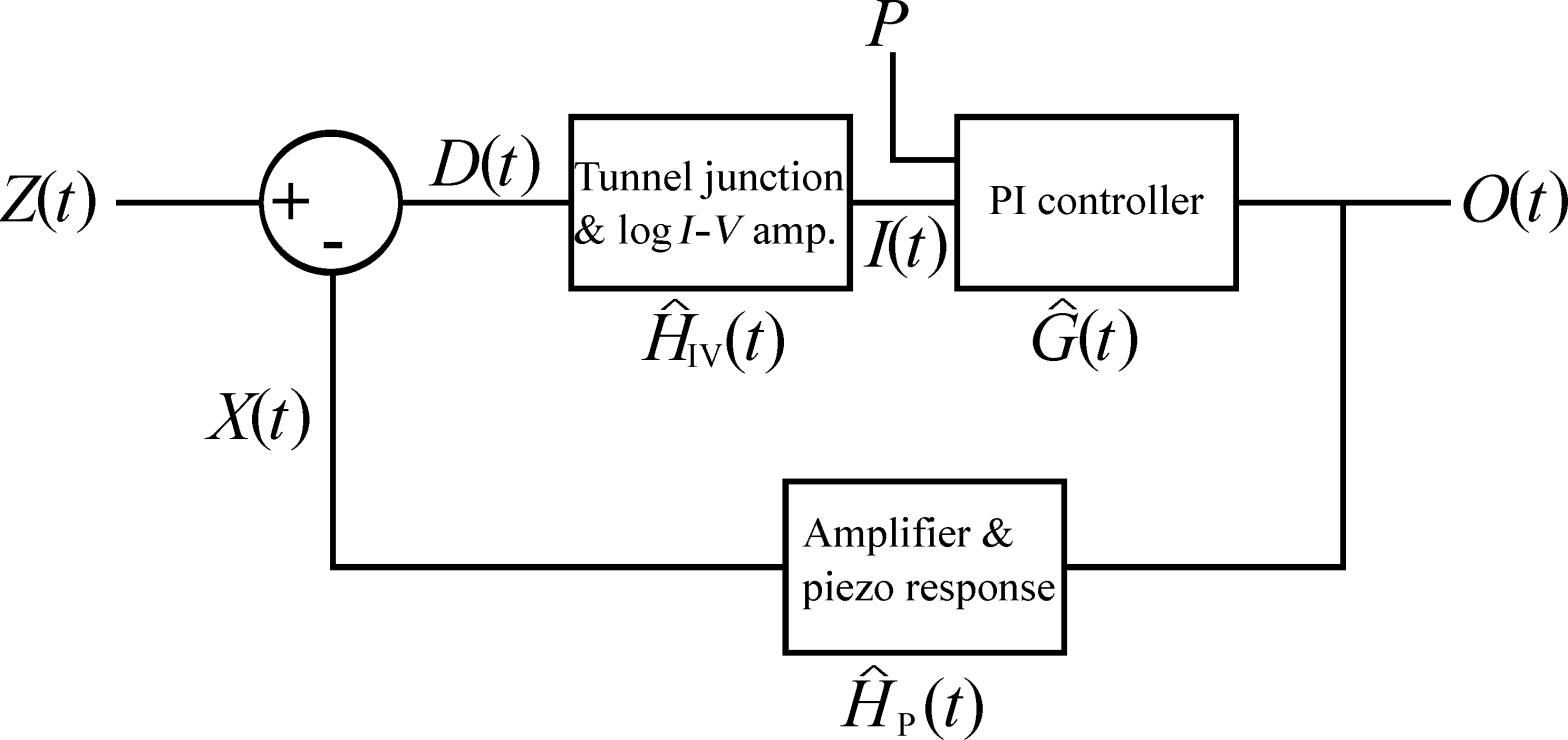
Schematic of an STM feedback loop. *Z*(*t*) and *X*(*t*) represent the sample height and *z*-piezo extension at time *t* respectively, and *P* is the set-point current. Other SPM systems can be modelled using the same feedback system by replacing the operator 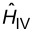, with an operator that describes the tip–sample interaction and signal amplification of the SPM to be modelled.

Before running simulations of our simplified SPM system we will first derive the model for the full SPM feedback system, and then set the transfer functions of unmodelled components to unity, to reduce the possibility for errors following their introduction. To avoid unnecessary generalisations we will discuss the feedback loop as it applies to the scanning tunnelling microscope (STM). The results are, however, equally applicable to other forms of SPM. For analysis of the full feedback loop of an STM (Figure 3) we start by considering that at any time *t* the tip will be above a particular area of the sample with height *Z*. Thus, the tip encounters the topography as a of the time *Z*(*t*). By using the extension of the z-axis of the piezoelectric scanner (*z*-piezo), *X*(*t*) (note that when modelling a complete SPM *X*(*t*) is no longer simply the output of the PI controller, as described in Equation 4), we can express the tip–sample distance, *D*(*t*), as

[5]
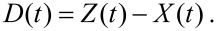


The measured tunnelling current is a function of the distance *D*(*t*), and also of the properties of the current-to-voltage (*I*–*V*) amplifier of the STM. As the tunnel current depends exponentially on the tip–sample distance the logarithm of the tunnel current is used for the feedback to improve the linearity of the feedback response. We shall refer to this log tunnel current as

[6]
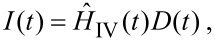


where 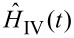 is the time dependent operator fully describing the tunnel junction, the *I*–*V* amplifier, and the logarithm operation.

The feedback controller then compares *I*(*t*) with a set-point, *P*, and tries to correct for discrepancies by modifying the output, *O*(*t*), to the *z*-piezo. We can write the feedback controller as the time-dependent operator 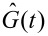, and hence

[7]
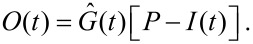


Finally, we can link the *z*-piezo extension to the feedback controller output with an operator, 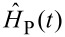. This describes both the high voltage amplifier use for the piezoelectric actuator and the mechanical response of the *z*-piezo itself:

[4]
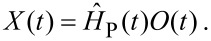


As the set-point acts as only a linear offset to the system we can set *P* = 0. Thus, combining Equation 6 and Equation 7 under this condition we get

[8]
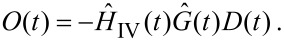


Combining this with Equation 4 gives

[9]
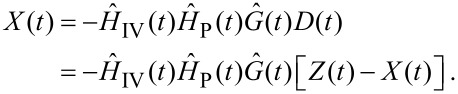


Here we apply a Laplace transform so that the transfer functions of the feedback components can be easily combined. This gives

[10]
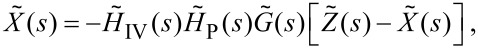


where 
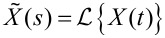
 and 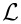 is the Laplace transform. Some minor rearrangement gives

[11]
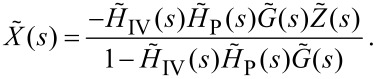


We are interested, however, in the output signal to the *z*-piezo, not its physical extension, as this is what the SPM controller records for the image. By simply considering the Laplace transform of Equation 4 (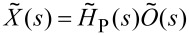) we arrive at a final result of

[12]
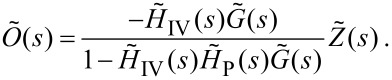


For this paper we are working in arbitrary units. Thus, the simulation needs to provide the relative response to change in gain settings rather than a response in physical units. Thus, we can set 
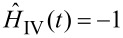
 (
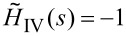
) as the logarithm should cancel the exponential dependence of the tunnel junction, and the gain of the *I*–*V* amplifier is simply linear, which is irrelevant if we are working in arbitrary units. To specifically consider the effect of the bandwidth of the SPM pre-amplifier, the functional form of 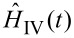 must be considered in more detail. More detail on modelling of such electrical components is given in the final section. Under this condition we can simplify Equation 12 to

[13]
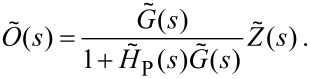


By applying the same argument used to derive Equation 3 we can write the operator for the PI-controller acting on an arbitrary function *f*(*t*) as

[14]
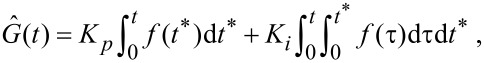


and thus in *s*-space this becomes

[15]
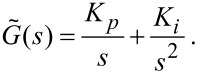


### Feedback performance without mechanical modelling

Initially we will study the stability of the STM feedback without modelling the mechanical resonances of the SPM system. For this we can substitute 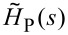 = 1 and Equation 15 into Equation 13. The feedback behaviour has been studied for four simulated surfaces:

[16]
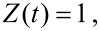


[17]
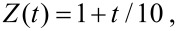


[18]
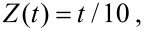


**Figure 4:**
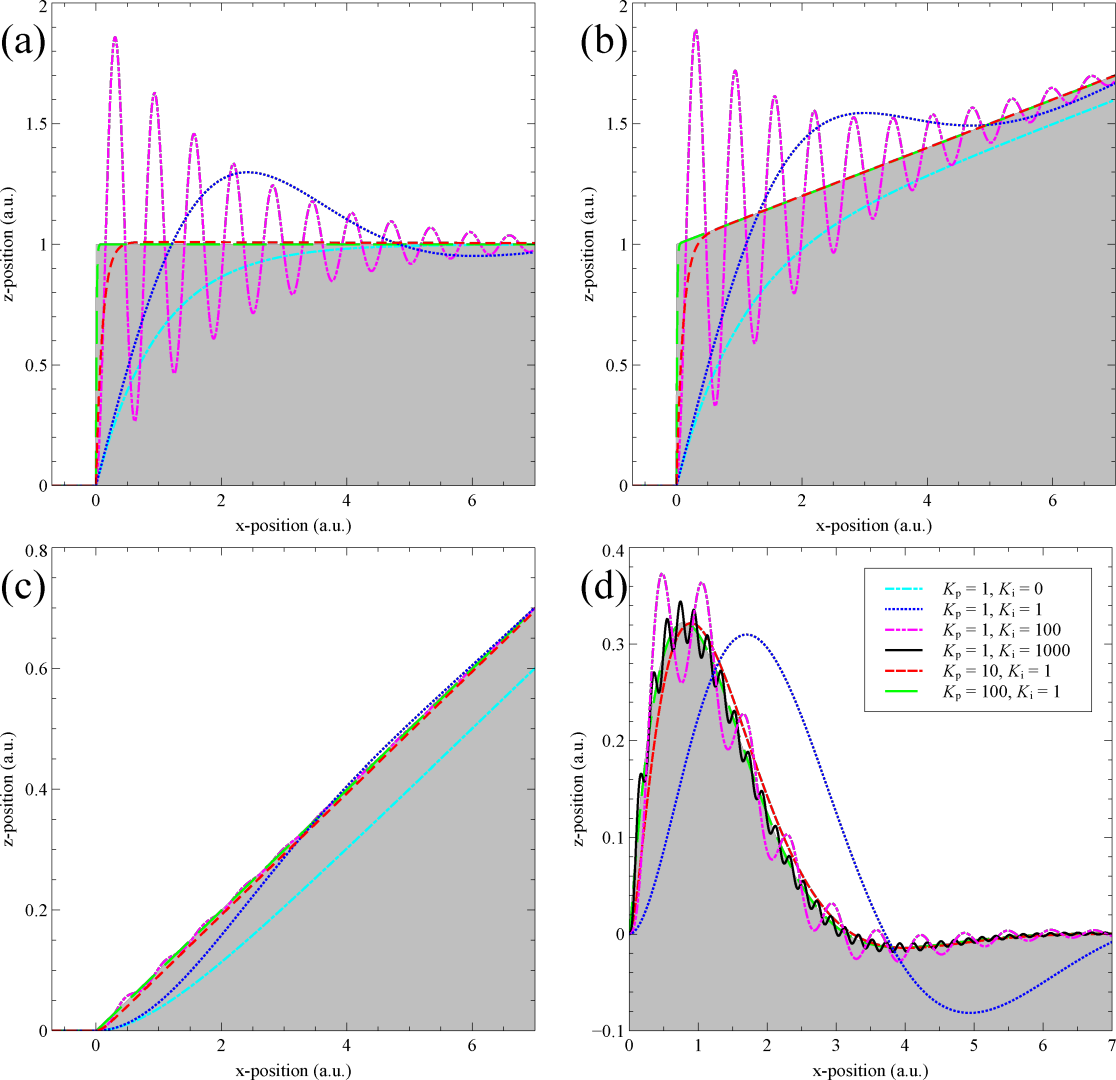
The feedback response of an SPM, without the inclusion of mechanical resonances, calculated for four different topographies, and for a range of feedback gains. Topographies in (a)–(d) correspond to Equation 16–Equation 19 respectively. Not all gains are plotted for all topographies to avoid overcrowding.

[19]
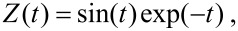


which correspond to a unit step, a ramp added to a unit step, a ramp, and a smooth topographical feature respectively. The results for a range of different feedback parameters are plotted in Figure 4. As the system is modelled in arbitrary units, time and *x*-position are equivalent if the tip is moving at a constant speed in *x*. It is clear from Figure 4 that the system behaves as expected. Steady state offsets appear for proportional only controllers if there is a *z*-ramp present, but is corrected by an integral controller.

When discussing the stability of the system, qualitatively one can see that tracking is maintained for a wide range of proportional and integral gains. For large integral gains the system oscillates, as expected. For all plotted gains oscillations always ring-off, never resulting in positive feedback. To further investigate the stability in the case of the unit step (Equation 16) the full system output in *s*-space can be analysed for poles. The final output in *s*-space is:

[20]
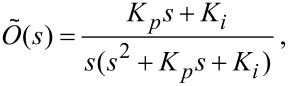


which results in three poles:

[21]
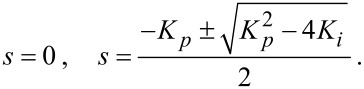


From this it is clear that if *K_p_* and *K_i_* are always positive (true for a feedback loop) no pole ever has a positive real value, and thus the system is always stable. We can also calculate that the feedback output will not oscillate if 
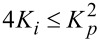
.

### Feedback performance with mechanical and electrical modelling

For a more realistic model of SPM feedback one should also model the response of electrical and mechanical components. Equations for such extra components should be tested individually and added sequentially to reduce the possibility of error as equations in *s*-space are rarely intuitive. To build up a full electrical and mechanical model of an arbitrary system is of little use when discussing stability as the system becomes too complicated to analytically derive the poles. Instead the above equations should be used in conjunction with real physical values from a SPM system to understand its stability.

As an example we will include a mechanical resonance for the *z*-piezo relative to its equilibrium position at its input voltage

[22]
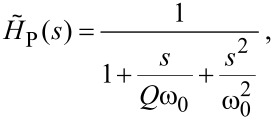


where *Q* is the quality factor of the resonance and ω_0_ is the angular eigenfrequency. It is important to note that this equation differs from that used in Reference [9], as this text mistakenly uses the mechanical response to a force rather than to a coupled mechanical offset. It is possible to model the transfer function of the *z*-piezo for an input voltage by replacing the numerator with the relevant piezoelectric coefficient. This is not done as it has no effect for a model in arbitrary units, and also as in this form Equation 22 can equally be used as the response of an AFM cantilever. It is, however, important to note that for some geometries of piezoelectric scanners, such as the tube scanner, the motion of the principle eigenmode is perpendicular to the *z*-axis [14], and thus cannot be included into our one dimensional model.

Substituting Equation 22 and Equation 15 into Equation 13, along with the equation for a unit step, the response of the full system in *s*-space is given by

[23]
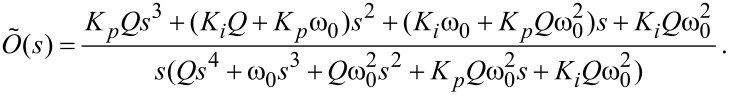


As the denominator is fifth order there are five poles. One pole at *s* = 0 shows the final response to the step. The functional form of the other four poles is too long to be qualitatively useful. However, the trend in pole positions can be qualitatively understood. Two poles correspond to the ringing oscillations from the system without the mechanical resonance, though the frequency and decay times are affected by the modelled resonance. Two further poles represent the excitation of the mechanical resonance. These poles can move into the unstable region if excited by high gains. The system can be made stable under higher gains by increasing the eigenfrequency or decreasing the *Q* of the resonator. For these reasons components with a high quality factor and a low resonant frequency are unsuitable as part of the SPM scanners.

**Figure 5:**
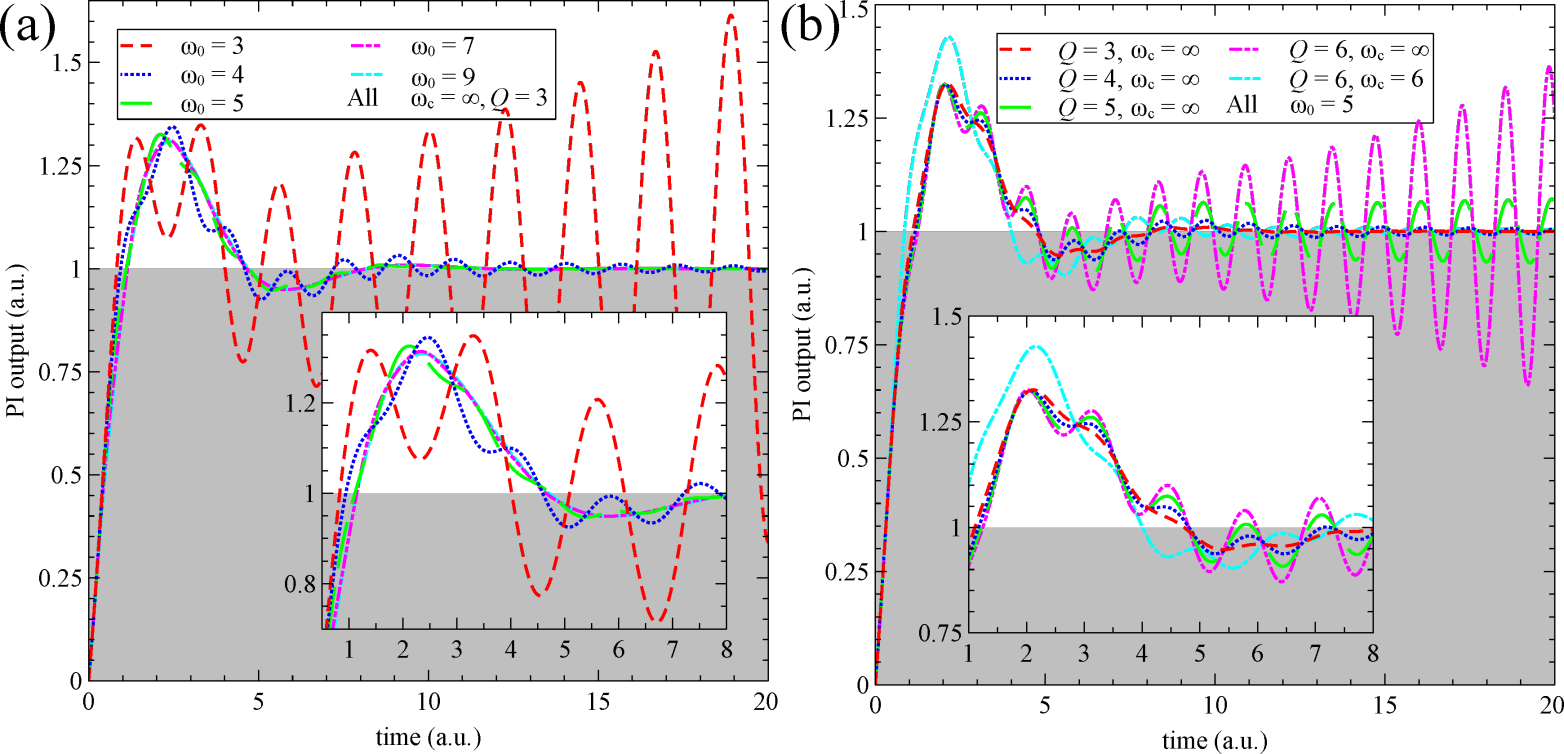
The feedback response of an SPM, including mechanical resonance. (a) Shows the evolution of the feedback output for varying eigenfrequency of the mechanical resonance. The stability improves for increasing resonant frequency. For all plots the bandwidth of the HV amplifier is infinite and the *Q* of the resonance does not vary. (b) Shows similar evolution in feedback output for varying *Q* of the resonance at a constant eigenfunction, with lower *Q* values stabilising the output. The cyan line shows the same resonance properties as the pink line, however by limiting the bandwidth of the HV amplifier to near that of the resonance, the stability is improved significantly. Both insets are zooms of the most important region of their respective plots.

In Figure 5a the PI controller output for a range of mechanical eigenfrequencies with a constant quality factor is plotted against time. Arbitrary units are used for both time and the PI output as the evolution under increasing eigenfrequency is valid for any magnitude. The *y*-axis is labelled PI output, not extension, as these are no longer equivalent when mechanical resonance is modelled. For all plotted outputs the bandwidth of the high voltage (HV) amplifier driving the *z*-piezo was assumed to be infinite, and hence Equation 22 was used without modification.

The evolution of the output under varying *Q* of the mechanical resonance is shown in Figure 5b. Again, in agreement with the polar analysis, the stability increases for lower *Q*. For higher *Q* the resulting instability can be diminished or eradicated by reducing the bandwidth of the HV amplifier. The transfer function of an amplifier with a finite bandwidth can be accurately modelled as a first order low-pass filter [15]

[24]
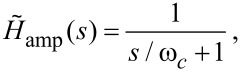


where ω*_c_* is the cut-off angular frequency (3-dB point) of the amplifier. As we are working in arbitrary units this amplifier has a gain of 1, the numerator of the transfer function can be replaced with the desired gain if needed. Including this, the full transfer function of the amplifier and piezo becomes

[25]
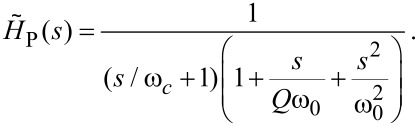


The cyan line in 5b shows the significant improvement in stability resulting from a cut-off frequency just above that of the mechanical eigenfrequency. This, however, comes at the cost of an increased overshoot. One also must be careful not to lower the cut-off frequency below the resonance, nor to used an over-damped (*Q* < 1/2) mechanical component as this can introduce a significant phase lag, causing new instabilities. The MATLAB code used to generate the data for Figure 4 and Figure 5 is included as Supporting Information File 1. This can be used to further explore the parameter space of the SPM PI controller.

The only component in Figure 3 that is not modelled, is the tunnel junction and the logarithmic amplifier, 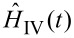. Considering the tunnel junction as an exponential decay with distance produces a current that is first amplified by an *I*–*V* preamplifier with a finite bandwidth. The logarithm of this output voltage is then taken either by a logarithmic amplifier or calculated numerically by the SPM controller. This results in a functional form for the time-domain operator action on the tunnel gap *D*(*t*) being

[26]
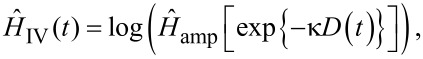


where κ is the characteristic decay length of the tunnel junction, and 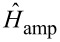 is the time-domain operator corresponding to the transfer function in Equation 24.

To calculate the *s*-space transfer function of Equation 26, one would need to calculate the Laplace transform of the exponential of an arbitrary function *D*(*t*). This may be possible for the specific functional forms of *D*(*t*) but is not generally applicable. One can approximate 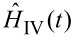 under the approximation that the logarithm and 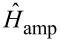 commute:

[27]
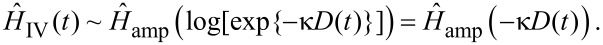


In arbitrary units, κ can be ignored and the transfer function of the tunnel junction approximates to 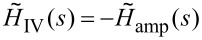. Under this approximation we ignore the effect of higher harmonics of frequencies present in *D*(*t*) being generated by the exponential dependence in the tunnel junction.

## Conclusion

We have derived an appropriate updated model to understand SPM feedback in the context of control theory. This model shows the intrinsic stability of the SPM feedback controller in an ideal environment. We further discuss methods to include modelling of mechanical resonances showing low frequency and high *Q* components to cause instabilities. By introducing amplifiers with bandwidths just above the mechanical eigenfrequency these instabilities can be controlled. The method presented here uses arbitrary units to show a generalised approach. The equations presented, however, can be used with real parameters from SPM systems to understand and model performance under a range of conditions.

## Supporting Information

File 1 MATLAB code used to simulate the presented feedback model.
